# Mortality and Loss to Follow-Up Among HIV-Exposed Infants After Option B^+^ Guideline Implementation in Amhara Regional State Referral Hospitals, Ethiopia

**DOI:** 10.3389/fped.2021.591963

**Published:** 2021-11-10

**Authors:** Mesfin Wudu Kassaw, Ayele Mamo Abebe, Biruk Beletew Abate, Mikiyas Amare Getu, Ayelign Mengesha Kassie

**Affiliations:** ^1^Department of Nursing, College of Health Science, Woldia University, Woldiya, Ethiopia; ^2^Department of Nursing, College of Health Science, Debre Berhan University, Debre Berhan, Ethiopia

**Keywords:** exposed infants, referral hospitals, Amhara region, loss to follow-up, mortality

## Abstract

**Background:** Prevention of mother-to-child transmission of HIV program (PMTCT) is a comprehensive approach that aimed for the wellbeing of all HIV-infected women, to prevent new HIV infection among infants born to HIV-positive mothers, and providing management for HIV-positive women and infants. Nevertheless, there was considerably high attrition within the prevention of mother-to-child transmission programs that was merely because of loss to follow-up (LTFU) followed by mortality. In resource-limited countries, one-third of infected children die before 1 year, and more than half of them die before 2 years. The aim of this study was to assess the prevalence or incidence of mortality and LTFU among infants born from HIV-positive mothers in the Amhara regional state referral hospitals, Ethiopia.

**Methods:** This study was conducted in five Amhara regional state referral hospitals' prevention of mother-to-child transmission departments. A simple random sampling technique with proportional allocation was used to assess the outcomes of 221 exposed infants. A retrospective cohort design was used in selecting the 221 exposed infants' document from the referral hospitals of the region, Amhara. The exposed infants' profiles were documented between January 1, 2014 and May 30, 2017.

**Results:** This study described attritions (death and loss-to-follow-up) of exposed babies in PMTCT departments of Amhara regional state referral hospitals in Ethiopia. In this study, low LTFU with zero death was reported. Residence, immunization status of babies, and place of delivery were independent factors of LTFU.

**Conclusions:** The cumulative incidence of mortality in this study was zero. This assured that the recommended option is substantial for the elimination of HIV-caused death in 2030 as per WHO plan. However, the cumulative incidence of LTFU was not zero.

## Background

Prevention of mother-to-child transmission program is a comprehensive management approach aimed for the well-being of all HIV-infected women, prevention of new HIV infection among infants born to HIV-positive mothers, and provision of management for HIV-positive women and infants (1). However, there was considerably high attrition within the PMTCT programs that was merely because of LTFU followed by mortality. For example, studies done in Africa reported that the total magnitude of LTFU at the PMTCT departments varied from 36.9 to 68.4% (2–4). The cumulative losses in sub-Saharan African PMTCT departments were also estimated with a range from 20 to 28% during antenatal care, up to 70% at 4 months postpartum, and close to 81% at 6 months after birth (5–7). The national rate of loss to follow-up (LTFU) from Kampala, Uganda revealed that the incidence of LTFU was 30.3% (95% CI: 28.4%, 32.3%) during option A and 28.4% (95% CI: 26.2, 30.7) during Option B+ (8). LTFU inhibits optimal outcomes of women enrolled to the PMTCT programs. Some studies revealed that LTFU within the PMTCT departments in resource-limited settings vary widely (2, 9–14) that range from 11 (14) to 75% (12). This means that the impact of LTFU would be proportional with the magnitude of losses. Although there are sufficient studies on a PMTCT program in Africa that reported a highly heterogenic data across the continent, there is also a meta-analysis on the region. This meta-analysis reviewed in 2013 reported that the pooled rate of LTFU among antenatal registration and delivery was 49.1%, and LTFU for infants within 3 months of delivery was 33.9% (15). Irrespective of the effort given for exposed infants such as PMTCT, the incidence of LTFU is consistently high. Moreover, these high incidences of LTFU were mentioned as a challenge for underestimation of mortality (16). The study from Uganda reported that monitoring adherence and retention of mothers on Option B+ were a big challenge. The information had also shown that poor adherence and retained mothers were substantial factors of LTFU (17). Unfortunately, variables that have associations with LTFU may also contribute to MTCT (3). From a study, a transport cost of above $2.75, waiting time of >1 h, and perception that the child is already infected were associated with LTFU positively. However, the mother's knowledge that the prophylaxis works effectively was protective for LTFU (17). Beyond a significant LTFU at the PMTCT departments, HIV/AIDS is the leading cause of mortality among women of reproductive age and contributes a great deal to the death of infants and children (1). In a resource-limited country, one-third of infected children die before 1 year, and more than half die before 2 years (18). Other studies revealed that the rate of mortality on HIV-positive infants before 6 months was 20%, and 35 to 40% die before 12 months of their life (19, 20). The rate of mortality among children in Thailand was 16 (16%) from a data reviewed between August 2014 and December 2015 (1, 21). A study from Uganda also reported that the cumulative incidence of death was 0.9% (95% CI: 0.5%, 1.5%) at option A and 1.4% (95% CI: 0.8%, 2.2%) at option B^+^ (8). Another study from Kigali, Rwanda reported an estimated 24 months rate of mortality among HIV-exposed infants as 4.8% (95% CI 3.3–6.9) (13). Some other studies had shown that child mortality increases considerably in children who were infected with HIV *via* their mothers (22, 23). The aim of this study was to assess the cumulative incidence of LTFU and mortality among HIV-exposed infants in the high standard healthcare provider health facilities after option B^+^ guideline implementation in Amhara regional state referral hospitals. Although the PMTCT program is rigorous to prevent both mortality and LTFU, there is a scarcity of data on the long-term effectiveness of the option B^+^ guideline either on decreasing LTFU or mortality through the duration of breastfeeding and on completing 24 months follow-up.

## Objectives

### General Objective

✓ To assess the cumulative incidence of LTFU and mortality, and identify factors associated with it among exposed infants after implementing option B^+^ guidelines in Amhara regional state referral hospitals, Ethiopia

### Specific Objectives

✓ To assess the cumulative incidence of LTFU among exposed infants after implementing option B^+^ guidelines in the Amhara regional state referral hospitals.
✓ To assess factors of LTFU among exposed infants after implementing option B^+^ guidelines in the Amhara regional state referral hospitals.
✓ To assess the cumulative incidence of mortality among exposed infants after implementing option B^+^ guidelines in the Amhara regional state referral hospitals.

## Methods

### Study Areas, Design, and Period

A retrospective cohort design was used in all of the referral hospitals of Amhara regional state. Amhara region is one of the administrative states in Ethiopia. The region is located to the south west of Ethiopia and has 15 administrative zones. The region has only five referral hospitals that provide better PMTCT services in accordance with the national guideline (24). Those referral hospitals are found in the north Shewa zone (Debrebirhan referral hospital, Debrebirhan, Ethiopia), the south Wollo zone (Dessie referral hospital, Dessie, Ethiopia), north Gonder zone (Gonder referral hospital, Gonder, Ethiopia), the west Gojjam zone (Felegehiwote referral hospital, Bahirdar, Ethiopia), and the east Gojjam zone (Debremarkos referral hospital, Debremarkos, Ethiopia). The period of data collection was from March 21 to May 18, 2019, from mothers' and infants' medical records.

### Cohort Definition

HIV-positive mothers who were pregnant and enrolled to the PMTCT departments between January 1, 2014 and May 30, 2017 and their children also enrolled to the program.

### Study Population

HIV-exposed infants and their mothers enrolled to all of the referral hospitals of the Amhara region between January 1, 2014 and May 30, 2017.

### Eligibility Criteria

HIV-exposed infants who had deoxyribonucleic acid polymerase chain reaction (DNA-PCR) test before 6 weeks of age and PCR or rapid antibody test result after 15 months old as per the Ethiopian guideline of PMTCT (25) were included, whereas infants who are on pediatric ART but their mothers were not enrolled to the PMTCT program and exposed infants transferred from other clinics or transferred out to other clinics were excluded. Children who dropped out the PMTCT program without DNA PCR test or RDT and mothers who dropped out before delivery were also excluded.

### Sample Size Determination

The samples size, 221, was calculated using a single population proportion formula. The following assumptions were considered in calculating the sample size: CI = cumulative incidence of LTFU after enrollment (15.4%) (26), [*Z*_α/2_ = *Z* score of 95% CI, *d* = margin of error (5% precision), and 10% incomplete data] (27).

### Sampling Techniques and Procedures

The sampling technique used was simple random sampling with proportional allocation to the study population. The sampling frame was exposed infants, who had complete documentation between January 1, 2014 and May 30, 2017 in the five Amhara regional state referral hospitals. Of these study population, 221 samples were drawn out and proportionally allocated to all of the five hospitals as per the number of clients enrolled to the PMTCT departments. On proportional allocation, 61 were allocated to Gonder referral hospital, 53 to Felegehiwot referral hospital, 26 to Debremarkos referral hospital, 54 to Dessie referral hospital, and 23 to Debreberhan referral hospital.

### Data Collection Tools

The cumulative incidence of LTFU and mortality were computed using a piloted data collection checklist. The checklist was piloted using 21 medical records of both infants and mothers from all of the five hospitals. The hospitals recorded the PMCT data routinely using the national manual PMCT registry form. The registry assures data quality and similarity across hospitals but sometimes there are personal errors because of professional integrity.

### Study Variables

The outcome variables of this study were LTFU or mortality, whereas the independent variables were socio-demographic status of mothers, obstetrics and gynecologic history of mothers, maternal viral and nutritional status, neonatal and infantile factors, and healthcare services provided to children and mothers.

### Definitions

**Exposed infant:** An infant or child born from an HIV-positive mother and/or on breastfeeding (28).

**Option B**^**+**^ **strategy:** A new WHO program to prevent mother-to-child transmission of HIV. All HIV-positive pregnant and breastfeeding women gave lifelong antiretroviral therapy irrespective of their CD4 count and clinical stage of the disease (28).

**LTFU:** If there are three failed attempts to track the infants after the last clinic visit or if 6 months elapsed since the infant was last seen at the clinic and recorded as LTFU on the infant card.

**Mortality**: A death of an infant after enrollment to the PMTCT department and the death recorded on the card of the child.

### Data Presentation and Analysis

The collected data were checked before entry to the epi-data manager. The data were entered to epi-data version 4.2.0.0 and transferred to SPSS version 24 for further analysis. Chi-square or Fisher exact test (*n* < 5) was used to assess the association between the independent variables and the outcome variable. A 95% confidence interval (CI) and a significant level (*p*-value) of <0.05 were used to assess the associations. The statistical tests were two sided and the level of statistical significance was set to be <0.05. A cumulative incidence was used to present mortality and LTFU during the first 24 months of life after enrollment to the PMTCT departments.

## Results

### Maternal Socio-demographic Status

Of the included 221 mother-child pairs, 217 documents were completed and reviewed. However, four documents were excluded because of incomplete data. Thus, the response rate of this study was 98.2%. The minimum and maximum ages of mothers enrolled to the PMTCT program were 20 and 41 years old, respectively. The lowest year that mothers existed with HIV was 1 year and the highest was 18 years. The mean and standard deviation of parity and gravidity status of mothers were 1.61 ± 0.94 (x ± sd) and 2.48 ± 1.25 (x ± sd), respectively. The minimum age of maternal gestation was 32 weeks and the maximum was 42 weeks (Table 1).

**Table 1:** Socio-demographic status of HIV positive mothers enrolled to option B^+^ PMTCT program in the Amhara regional state referral hospitals, Ethiopia.

**Variables**	**Categories**	**Frequency (*n* = 217)**	**Percent**
Maternal education	Unable to read and write	26	12.0
	Able to read and write	56	25.8
	Primary education	73	33.6
	Secondary education	62	28.6
Occupation	Employed	82	37.8
	Not employed	135	62.2
Marital status	Married	159	73.3
	Divorce	17	7.8
	Separated	16	7.4
	Single	25	11.5
Place of delivery	Home	44	20.3
	Private health facility	13	6.0
	Public hospital	140	64.5
	Health center	20	9.2
Residence	Rural	76	35
	Urban	141	65
Maternal care provided	No care	27	12.4
	ANC 1 and 2	18	8.3
	ANC 2 and 3	9	4.1
	ANC 2, 3, and 4	82	37.8
	ANC 3 and 4	32	14.7
	Delivery	49	22.6
TT vaccination	TT1	42	19.4
	TT2	75	34.6
	TT3	51	23.5
	TT4	22	10.1
	TT5	8	3.7
	Not taken	19	8.8
Mode of delivery	Spontaneous vaginal	179	82.5
	Cesarean section	32	14.7
	Instrumental delivery	6	2.8
ART adherence	Good	125	57.6
	Poor	63	29
Entry to PMTCT	During ANC	73	33.6
	During PNC	66	30.4
	Prior to pregnancy	78	35.9
Maternal cotrimoxazole	Yes	46	21.2
	No	171	78.8
Stage of HIV	Stage 1 and 2	162	74.7
	Stage 3 and 4	55	25.3
Illness during pregnancy	Yes	35	16.1
	No	182	83.9
TB during pregnancy	Yes	9	4.1
	No	208	95.9
Disclosure status of HIV	Yes	124	57.1
	No	93	42.9
For whom disclosed	Husband	89	71.77
	Relative	12	9.68
	Sibling	2	1.61
	Friends	20	16.13
Opportunistic infection	Yes	15	6.9
	No	202	93.1
Type of OIs other than	PCP	5	2.3
TB	Recurrent URTIs	4	1.8
	Candidiasis	6	2.8
Iron foliate intake	Not taken	75	34.6
	Full dose for 3 months	95	43.8
	Partial dose taken	47	21.1

### Loss to Follow-Up and Mortality

The cumulative incidence of LTFU in the Amhara regional state referral hospital was 19 (8.8%), (95% CI, 5.4%−12.4%) among 217 exposed infants enrolled to the PMTCT department between January 2014 and 2017. The cumulative incidence of mortality in the region was zero.

### Factors Associated With Loss to Follow-Up

Residence (*p* ≤ 0.003), immunization status of children (*p* ≤ 0.004), and place of delivery (*p* < 0.005) had associations with the incidence of LTFU. The statistics indicated that mothers who were from rural areas, children who had incomplete immunization status, and mothers who gave birth at home were increasing the odds of LTFU (Table 2).

**Table 2:** *X*^2^-test to assess the associations with loss to follow up among HIV exposed infants on Option B^+^ PMTCT program in Amhara regional state referral hospitals.

**Variables**	**Categories**	**Outcomes of PMTCT (*****n*** **=** **217)**		***P***
		**LTFU (%)**	**Completed (%)**	
Residence	Rural	13 (17.1)	63 (82.9)	<0.05
	Urban	6 (4.3)	135 (95.7)	
Maternal cotrimoxazole	Yes	7 (15.2)	39 (84.8)	0.14
	No	12 (7.0)	159 (93.0)	
Occupation	Employed	3 (3.7)	79 (96.3)	0.07
	Not employed	16 (11.9)	119 (88.1)	
Immunization status	Fully immunized	9 (5.5)	156 (94.5)	<0.05
	Partially immunized	10 (19.2)	42 (80.8)	
	No	6 (20.7)	23 (79.3)	
Stages of maternal HIV	Stage 1 and 2	7 (4.3)	155 (95,7)	<0.001
	Stage 3 and 4	12 (21.8)	43 (78.2)	
Disclosure status of mothers	Yes	7 (5.6)	117 (94.4)	0.10
	No	12 (12.9)	81 (87.1)	
Opportunistic infections	Yes	0 (0)	15 (1,000)	0.37
	No	19 (9.4)	183 (90.6)	
Place of delivery	Home	9 (20.5)	35 (79.5)	<0.05
	Health facility	10 (5.8)	163 (94.2)	

## Discussion

The objective of this study was to assess LTFU and mortality from the time of enrollment to the completion of the PMTCT follow-up program. However, it did not consider the loss of mothers before giving birth and obtaining the first HIV test for their children. The cumulative incidence of LTFU in Amhara regional state referral hospitals was 8.8%, 95% CI (5.4–12.4%). The results of this study are higher than the report of a study conducted in England (29). The difference might be because of socio-demographic distinction. However, the incidence of LTFU in this study is lower than other studies conducted in Africa (2, 30–33). The variation might be due to a difference in a study time, since almost all the former studies studied before 6 years on average compared to the present study. This study has lower LTFU than a systematic review that considers the incidence of LTFU in sub-Saharan Africa (34). The present study's incidence of LTFU was also lower than the study that compared options A and B^+^ in Uganda (8). The difference might be because of socio-demographic or design difference. The current study was done using secondary data, but the former study studied prospectively. This study was lower than another national level study (20, 35). This might be because of the study period and population difference. The finding was lower than that of a study done in northwest Ethiopia, Woliso 39.4% (36), and a study done in Bishofitu Hospital, 22.2% (37). These differences might be because of either study period difference or difference in types of guidelines implemented at the PMTCT departments. Poverty, lack of paternal support, poor mobility, long distance beteween residence and care structure, and cost of transpot were factors for LTFU (1). In this study, rural residence (*p* < 0.005), incomplete immunization status (*p* < 0.005), maternal advanced AIDS status (stage 3 and 4) (*p* < 0.001), and home delivery (*p* < 0.005) were associated with LTFU using χ^2^ test. A study in Brazil reported that rural residence was associated with LTFU (33). Another study in Uganda (8) reported that HIV-infected mothers and their exposed children who had adequate health information have a lower risk of LTFU. A study in Western Kenya (31) and in Malawi (32) showed that HIV-exposed or -infected children who were also under nutrition were less likely to attend scheduled visits. Fortunately, the cumulative incidence of mortality after enrollment to the PMTCT program in this study area was zero. This study had a lower result than a study done in Uganda that reported 19% of mortality in 2006 (38). The difference might be because of guideline difference. The current study included mother-child pairs who were treated under the new version, option B^+^. However, the former paper was studied using the old version, option A, because, the new version has better healthcare services (1). This study has a lower mortality result than another study from Uganda that compared option A and option B^+^ programs. The former paper reported 0.9 and 1.4% mortality in option A and option B^+^, respectively (8). The difference might be because of study period difference. The current study was conducted for the data recorded between January 1, 2014 and May 30, 2017 but the former paper considers from 2010 to 2011 for option A, and from 2013 to 2014 for option B^+^. In addition, the former paper used a large sample size. The finding of this study contradicts plenty of research findings that reported that HIV-exposed infants have a higher risk of morbidity, mortality, growth compromise, developmental delays, and differences in immune development (39–45).

## Limitations and Strengths

### Limitations

Since the study included only those babies who had DNA PCR test or RDT test after 15 months, those without a test or with a test after 6 weeks and before 15 months will be missed, which may likely underestimate the LTFU proportion reported. The authors used proportional sampling techniques although this is a retrospective study design. The opportunity of using all available medical records has been missed. While the authors reported the study as using a cohort study design, the statistical analysis was not appropriate or enough for a cohort study. As PMTCT care requires regular follow-up visits, it is assumed that time to event data may be available. Incident rates rather than cumulative incidence as well as Cox-proportional hazard model or other longitudinal data analysis rather than bivariate analysis are preferred.

### Strengths

This study considers the latest option (option B^+^) to provide recommendation concretely for policymakers. In addition, it assess the highest standard of PMTCT care from the tertiary health facilities only, which is an indirect indicator of the region's PMTCT performance.

## Data Availability Statement

The raw data supporting the conclusions of this article will be made available by the authors, without undue reservation.

## Ethics Statement

This study's protocol was reviewed and approved by Woldia University Institutional Review Board. Written informed consent from the participants' legal guardian/next of kin was not required to participate in this study in accordance with the national legislation and the institutional requirements.

## Author Contributions

MWK, BBA, MAG, AMK, and AMA conceived the title and designed the study, conducted field study, analyzed the data, drafted and critically revised the article, and wrote the final manuscript. All authors have read and approved the final version of this manuscript and also agreed to be accountable for all aspects of the work.

## Funding

This study was sponsored by Woldia University, College of Health Sciences, Research and Development Office with a grant number 1678/RCVP/2018. The funder had no role in collecting, analyzing, or writing the paper.

## Conflict of Interest

The authors declare that the research was conducted in the absence of any commercial or financial relationships that could be construed as a potential conflict of interest.

## Publisher's Note

All claims expressed in this article are solely those of the authors and do not necessarily represent those of their affiliated organizations, or those of the publisher, the editors and the reviewers. Any product that may be evaluated in this article, or claim that may be made by its manufacturer, is not guaranteed or endorsed by the publisher.

## Abbreviations

AIDS: acquired immune deficiency syndrome
ANC: antenatal care
ART: antiretroviral therapy
ARV: antiretroviral
DNA: deoxyribonucleic acid
HAART: highly active antiretroviral therapy
HIV: human immune virus
LTFU: loss to follow-up
MTCT: mother-to-child transmission
NVP: nevirapine
PCR: polymerase chain reaction
PMTCT: prevention of mother-to-child transmission
PNC: postnatal care
TB: tuberculosis
UNICEF: United Nations Children's Fund
WHO: World Health Organization
DBS: dried blood spot
sdNVP: single-dose nevirapine
cART: combined ART.
